# Expression of Connective Tissue Growth Factor in Male Breast Cancer: Clinicopathologic Correlations and Prognostic Value

**DOI:** 10.1371/journal.pone.0118957

**Published:** 2015-03-04

**Authors:** Miangela M. Lacle, Paul J. van Diest, Roel Goldschmeding, Elsken van der Wall, Tri Q. Nguyen

**Affiliations:** 1 Departments of Pathology, University Medical Center Utrecht, Utrecht, The Netherlands; 2 Division of Internal Medicine and Dermatology, University Medical Center Utrecht, Utrecht, The Netherlands; Garvan Institute of Medical Research, AUSTRALIA

## Abstract

Connective tissue growth factor (CTGF/CCN2) is a member of the CCN family of secreted proteins that are believed to play an important role in the development of neoplasia. In particular, CTGF has been reported to play an important role in mammary tumorigenesis and to have prognostic value in female breast cancer (FBC). The aim of the present study was to investigate clinicopathologic correlations and prognostic value of CTGF in male breast cancer (MBC) and to compare these findings with FBC. For this, we studied CTGF protein expression by immunohistochemistry in 109 MBC cases and 75 FBC cases. In MBC, stromal CTGF expression was seen in the majority of the cases 78% (85/109) with high expression in 31/109 cases (28.4%), but expression in tumor cells was only seen in 9.2% (10/109) of cases. High stromal CTGF expression correlated with high grade and high proliferation index (>15%) assessed by MIB-1 immunohistochemical staining. CTGF expression in tumor epithelial cells did not correlate with any of the clinicopathologic features. In FBC, stromal CTGF expression positively correlated with mitotic count and tumor CTGF expression was associated with triple negative status of the tumor (p = 0.002). Neither stromal nor tumor epithelial cell CTGF expression had prognostic value in MBC and FBC. In conclusion, stromal CTGF expression was seen in a high percentage of MBC and was correlated with high grade and high proliferation index. In view of the important role of the microenvironment in cancer progression, this might suggest that stromal CTGF could be an interesting target for novel therapies and molecular imaging. However, the lack of association with prognosis warrants caution. The potential role of CTGF as a therapeutic target for triple negative FBC deserves to be further studied.

## Introduction

Connective tissue growth factor (CTGF/CCN2) is a member of the CCN family of six secreted matricellular proteins consisting of four conserved subdomains [1], and is expressed in several cell types, such as endothelial cells, fibroblasts and leukocytes but especially in osteoblasts and chondrocytes. It functions as a multi-functional signalling modulator involved in a wide variety of biologic and pathologic processes including control of proliferation, migration and adhesion, regulation of growth, development and differentiation, wound healing, regeneration and cell death [2–4]. CTGF has been reported to play an important role in mammary tumorigenesis and its expression has been proven to be associated with increased migration and angiogenesis [2]. Significant associations have been found between CTGF overexpression and stage, tumor size, lymph node status, age and prognosis in female breast cancer (FBC) patients suggesting that CTGF may play a role in the progression of breast cancer [5].

Male breast cancer (MBC), contrary to FBC, is rare and not well characterized. Currently, treatment regimens for MBC are based on the assumption that it is largely similar to its female counterpart, and that prognostic features and therapeutic targets of FBC can be extrapolated to MBC. Although there are indeed similarities between MBC and FBC, there is also mounting evidence that they are in fact biologically quite different [6–12]. At the same time, there is yet little evidence that prognostic features that have been established in FBC are valid for MBC as well. In the current study, we set out to investigate the expression, clinicopathologic correlations and prognostic value of CTGF in a relatively large group of MBC.

## Materials and Methods

### Patients and specimens

All consecutive cases of surgical breast specimens of invasive MBC from 1986–2011 were collected from 7 different pathology laboratories in The Netherlands (St. Antonius Hospital Nieuwegein, Diakonessenhuis Utrecht, University Medical Center Utrecht, Laboratory for Pathology East Netherlands) as described before [7–10]. Hematoxylin and eosin (HE) slides were reviewed by three experienced observers (PJvD, RK, MML) to confirm the diagnosis and assess tumor characteristics. The tumors were categorized by histological type according to the WHO [13]. Pathology reports were used to retrieve information on age, tumor size and lymph node status. For the MBC cases immunohistochemical CTGF data of 3 patients was lost due to poor core morphology. A total of 109 cases were included from which there was enough material left in the paraffin blocks to perform tissue microarrays and immunohistochemistry. For the FBC cases immunohistochemical CTGF data of 1 patient was lost due to poor core morphology. A total of 75 random FBC cases from one newly constructed TMA were used to compare the immunohistochemistry findings between MBC and FBC.

Since we used archival pathology material which does not interfere with patient care and does not imply the physical involvement of the patient, no ethical approval is required according to Dutch legislation [14]. Use of anonymous or coded left over material for scientific purposes is part of the standard treatment contract with patients and therefore informed consent procedure was not required according to our institutional medical ethical review board. This has also been described earlier by van Diest et al. [15].

### Immunohistochemistry

**Fig 1:**
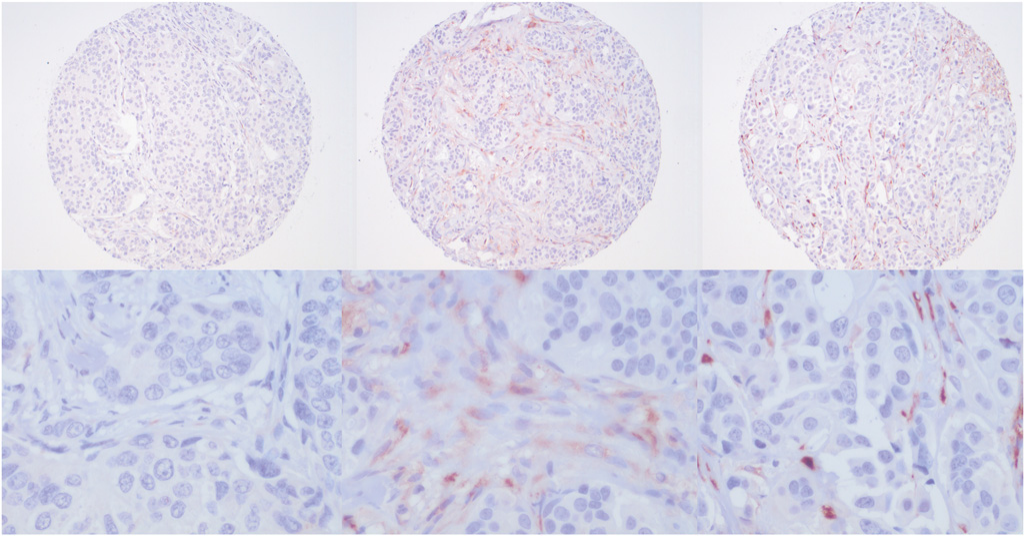
Representative low and high magnification examples of stromal CTGF expression in male breast cancer. Left: weak, middle: moderate, right: strong.

MBC tissue microarrays (TMAs) were constructed as described before [16] and four μm thick sections were cut from the TMA blocks. For FBC a previously constructed TMA was used. Immunohistochemical staining for CTGF (dilution 1:5000, Santa Cruz, goat anti-CTGF (L-20), sc-14939) was performed as follow: 20 minutes antigen retrieval with citrate buffer (pH 6) method followed by 60 minutes primary antibody incubation at room temperature. CTGF in stromal and tumor cells was scored by three experienced observers (TN, RG, ML). The intensity was quantified as weak (1), moderate (2) or strong (3) (Fig. 1). The percentage of positive stromal or tumor cells was given a score of 1 if less than a third were positive, 2 if between one and two thirds and 3 if more than two thirds were positive. For stromal cells, a combined score was calculated by adding intensity and percentage of positive cells which resulted in a score of 0 to 6. This score was then dichotomized into low (1–3) and high expression (4–6). ER, PR, HER2, HIF1α and Ki67/MIB1 data were derived from our previous study (9).

### Statistics

Information regarding prognosis and therapy was requested from the Integral Cancer Registration of The Netherlands (IKNL). Survival data was available for 100 (92%) cases. The mean follow up was 5.7 years (range 0.1–20.3 years). Therefore, survival analysis was based on 5 years survival rates.

Data analysis was performed using IBM SPSS statistics (version 20). Pearson’s chi-square or Fisher’s exact tests were used when appropriate. Survival rates were estimated using the Kaplan–Meier method and differences between curves were tested for significance using the log-rank test. All tests were two-sided and *p-*values less than 0.05 were considered significant.

## Results

### Patients and tumor characteristics

The clinicopathologic characteristics of the MBC and FBC patients are shown in Table 1. The age of the male patients ranged from 32 to 89 years (average: 66 years). Tumor size ranged from 0.4 to 5.5 cm (average: 2.1 cm). In 86.2% of patients the lymph node status was known by axillary lymph node dissection or sentinel node procedure and in 54.3% of these patients lymph node metastases were found. The majority of cases was diagnosed as invasive ductal carcinoma (90.8%). Most tumors were ER positive (92.7%) while PR and AR positivity was also common (65.1% and 77.8%, respectively). HER2 amplification was rare (3.7%). According to the modified Bloom and Richardson score 23.9% of the tumors were grade 1, 50.4% were grade 2 and 25.7% were grade 3.

**Table 1:** Baseline clinicopathologic features of 109 male breast cancers (MBC) and 75 female breast cancers (FBC).

*Characteristics M*	*MBC (*n = *109)*	*FBC (*n = *75)*
Age (mean), years	66	56
</ = 50	12 (11%)	27 (36%)
>50	97 (89%)	48 (64%)
Histological type		
Ductal	99 (91%)	61 (81.3%)
Lobular	2 (1.8%)	7 (9.3%)
Invasive cribriform	1 (0.9%)	0 (0%)
Mixed (ductal/lobular)	2 (1.8%)	5 (6.7%)
Mucinous	2 (1.8%)	1 (1.3%)
Papillary	1 (0.9%)	1 (1.3%)
Invasive micropapillary	1 (0.9%)	0 (0%)
Adenoid cystic	1 (0.9%)	0 (0%)
Tumor size (mean), cm	2.2 (n = 106)	2.8 (n = 74)
T1	53 (50%)	32 (43.2%)
T2	50 (37.2%)	34 (46%)
T3	3 (2.8%)	8 (10.8%)
Histological grade		
I	26 (23.9%)	6 (8%)
II	55 (50.4%)	26 (34.7%)
III	28 (25.7%)	43 (57.3%)
Mitotic index		
M1	47 (43.1%)	14 (18.7%)
M2	30 (27.5%)	21 (28%)
M3	32 (29.4%)	40 (53.3%)
Lymph node metastasis	n = 94	n = 73
Absent	43 (45.7%)	49 (67.1%)
Present	51 (54.3%)	24 (32.9%)

### CTGF expression and clinicopathologic correlations

In MBC, any CTGF expression (in stromal and/or tumor cells) was seen in 89/109 (81.7%) of cases. CTGF expression in tumor cells was seen in 10/109 (9.2%) of cases, and stromal cell expression in 85/109 (78%) of cases of which 31 (36.5%) showed high expression. Stromal cell expression of CTGF did not correlate with epithelial tumor cell CTGF expression.

**Fig 2:**
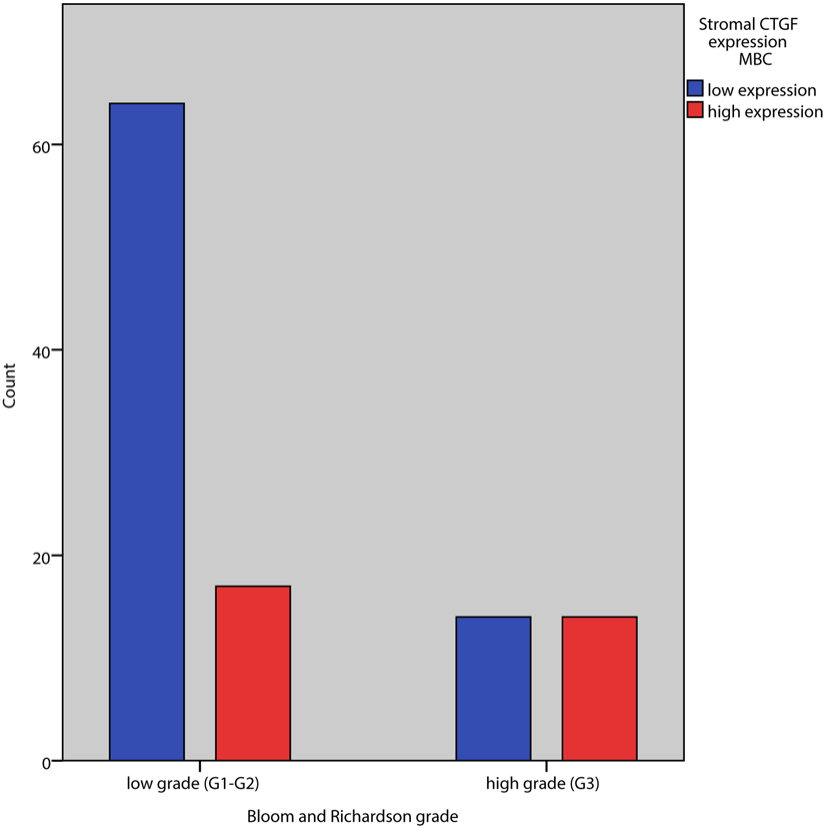
Association between tumor grade and CTGF expression in tumor stromal cells in male breast cancer.

**Fig 3:**
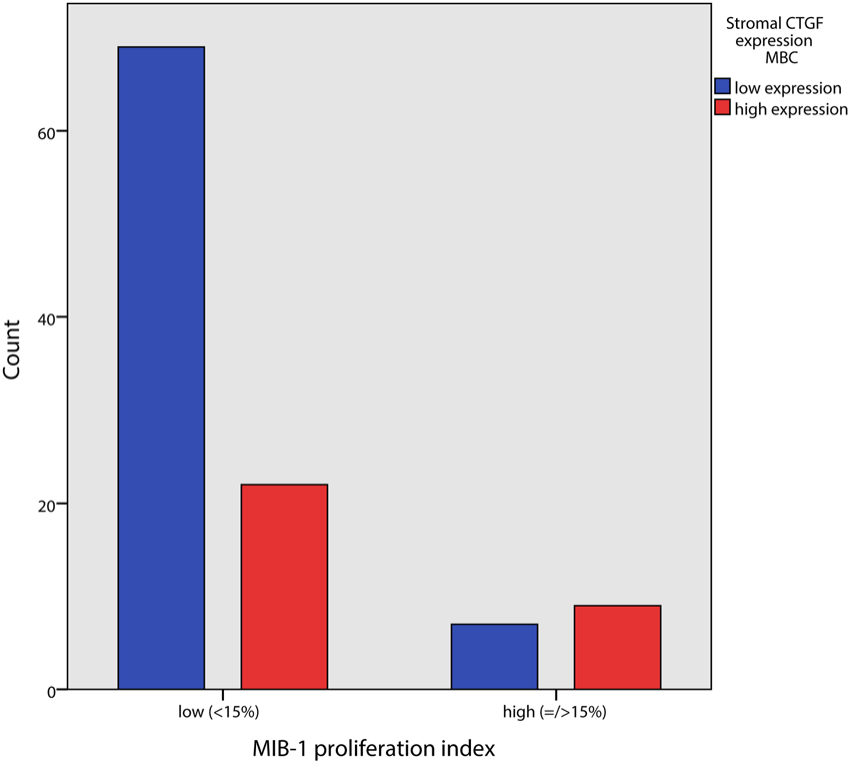
Association between MIB1 proliferation index and CTGF expression in tumor stromal cells in male breast cancer.

Stromal CTGF expression correlated positively with grade (p = 0.018) and high stromal expression even stronger with high grade (G3) versus grade 1/2 (p = 0.007). Of the three components of the Bloom and Richardson grading system, a high stromal CTGF expression positively correlated with gland formation and tumor nuclear pleomorphism (p = 0.005 and p = 0.006 respectively) but not with mitotic index in tumor epithelial cells. High stromal CTGF expression was positively correlated with high MIB1 proliferation index in tumor epithelial cells (= />15%) (p = 0.034) (Table 2, Figs. 2 and 3).

**Table 2:** Associations between stromal CTGF expression and clinicopathologic features in male breast cancer.

		*CTGF expression*		
Variable	*n*	*low*	*high*	P-*value*
Tumor size				*1*
T1	53	38	15	
T2/T3	53	38	15	
Grade				***0*.*003***
G1/G2	81	64	17	
G3	28	14	14	
Mitosis				*0*.*176*
M1/M2	77	58	19	
M3	32	20	12	
Gland formation				
G1/G2	55	46	9	***0*.*005***
G3	54	32	22	
Nuclear pleomorphism				
G1/G2	74	59	15	***0*.*006***
G3	35	19	16	
MIB				***0*.*009***
0–15	91	69	22	
>15	16	7	9	

**Fig 4:**
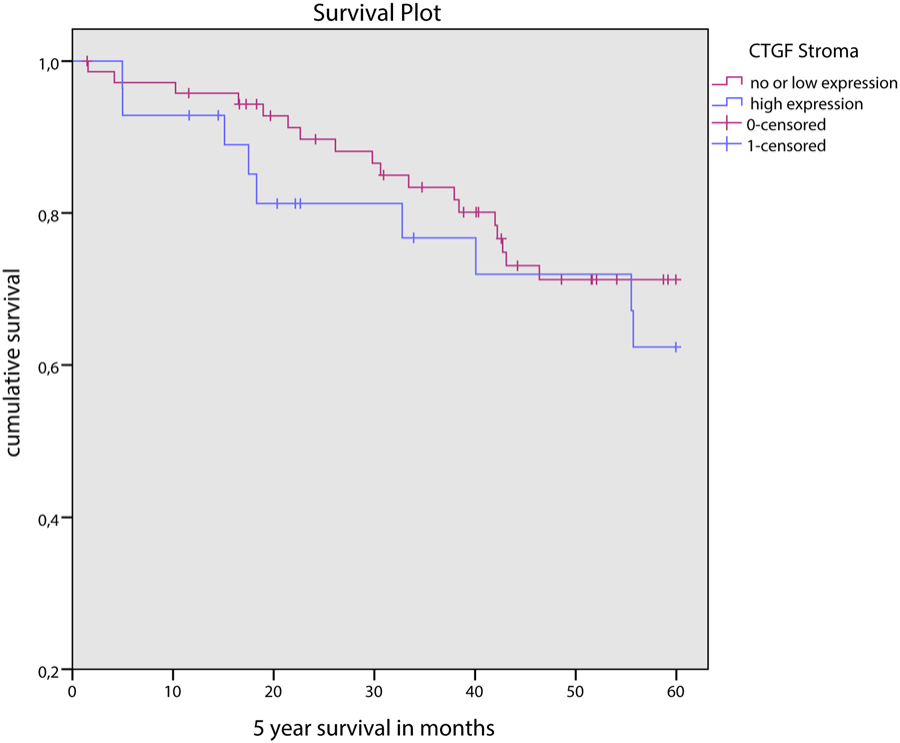
5 year survival curves for low and high stromal CTGF expression in male breast cancer.

Stromal CTGF expression did not correlate with other clinicopathologic features such as age, tumor type, tumor size, lymph node status or hormonal status, or HIF-1α expression. CTGF expression in tumor cells did not correlate with any of the clinicopathologic features mentioned above. In univariate survival analysis, stromal nor epithelial, nor combined stromal and epithelial CTGF expression was correlated with survival (Fig. 4).

**Fig 5:**
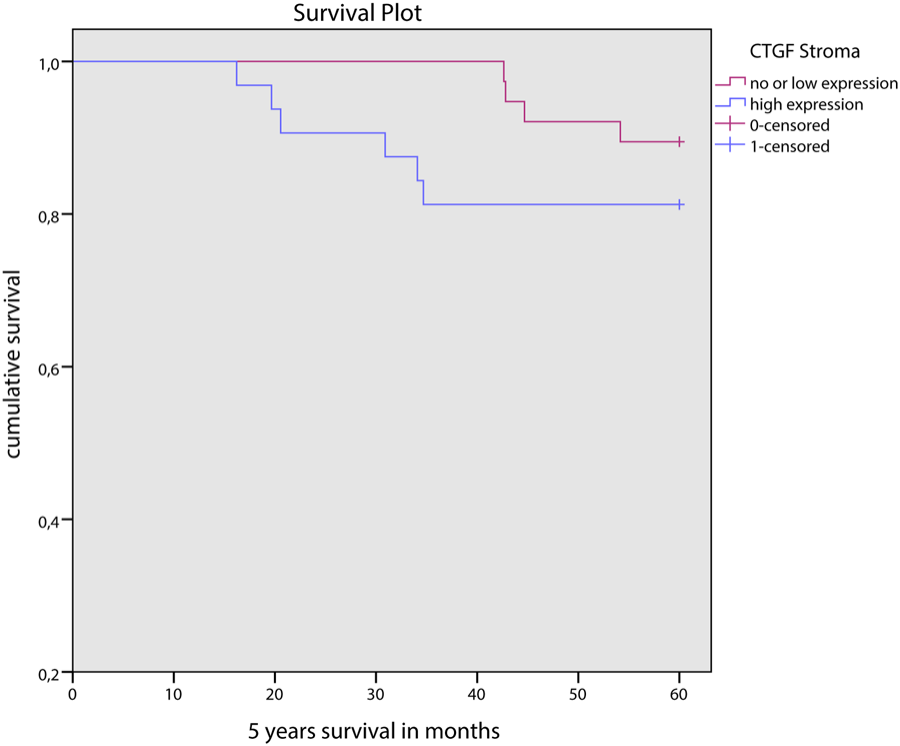
5 year survival curves for low and high stromal CTGF expression in female breast cancer.

In FBC, any CTGF expression (in stromal and/or tumor cells) was seen in 71/75 (94.7%) of cases, stromal cell expression in 69/75 (82%) of cases of which 35 (50.7%) showed high expression, and CTGF expression in tumor cells in 18/75 (24%) of cases. Stromal CTGF expression was correlated with triple negative status of the tumor (p = 0.002); all of the eight triple negative tumors showed stromal expression of CTGF, five of which had high stromal expression. Any stromal CTGF expression correlated positively with mitotic count (p = 0.010). Also for FBC, stromal nor epithelial, nor combined stromal and epithelial CTGF expression was correlated with survival (Fig. 5).

## Discussion

The aim of the present study was to investigate the prognostic value of CTGF in MBC, as well as evaluate clinicopathologic correlations, since CTGF was previously described to be an important player in carcinogenesis and a biomarker of aggressive FBC. Although most previous studies on CTGF used molecular techniques we chose to evaluate CTGF expression on the protein level by a previously validated immunohistochemical protocol [17], and compared expression to FBC.

Stromal CTGF expression was seen in the majority of MBC cases 78% (85/109) with even high expression in 31 of the 109 cases (28.4%). However, CTGF expression in tumor cells was seen in only 9.2% (10/109) of the cases. This is in keeping with a previous report where CTGF expression was observed only in fibroblasts of mammary tumors and not in tumor epithelial cells [18]. The latter report suggested that CTGF is produced in the stromal fibroblasts under influence of TGF-β1 which in turn is produced by mammary tumor epithelial cells. A more recent study by Caparelli et al. proposed a compartment specific role for CTGF for tumor formation in breast cancer showing that overexpression of CTGF by tumor epithelial cells leads to tumor cell digestion and inhibition of tumor growth. On the other hand, overexpression of CTGF in fibroblasts changes the tumor microenvironment and fuels anabolic tumor cells, but drives the induction of autophagy and a senescence phenotype in fibroblasts, which may further promote tumor growth [19].

Stromal CTGF correlated with high grade and high proliferation as measured by MIB1 index. This is in line with previous studies in hepatocellular carcinoma showing that CTGF contributed to dedifferentiation and growth [20, 21]. Our results point to a similar mechanism, but further studies are necessary to unravel the mechanisms by which stromal CTGF expression affects tumorigenesis in MBC. There was no correlation to mitotic index, another proliferation marker. This may be explained by the fact that although mitotic index and MIB1 index are both proliferation markers which are usually correlated, they describe different features of proliferation, mitotic index reflecting the number of cells actually in the mitotic phase, while MIB1 stains all cycling cells. Since the duration of the different phases of the cell cycle is quite variable in tumors, the correlation between mitotic index and MIB1 index is not absolute.

There was no significant correlation between CTGF stromal expression and HIF-1α expression, in contrast with Caparelli et al. who demonstrated that the metabolic promotion of tumor growth in breast cancer cells by CTGF is through activation of HIF-1α. These results may suggest that the mechanism by which CTGF promotes tumor growth in MBC is not HIF-1 mediated.

Stromal CTGF expression did not correlate with other studied clinicopathologic features such as age, tumor type, tumor size, lymph node status, hormone receptor or HER2 status. This is at variance with previous findings in FBC where overexpression of CTGF on a molecular level was reported to be significantly associated with tumor size, lymph node status and HER2 [5]. In our study expression of stromal CTGF on a protein level in FBC did correlate with mitotic count but not with other clinicopathologic features including grade, tumor size and lymph node status. Epithelial cell CTGF expression was not correlated with any of the studied clinicopathologic features, suggesting that the mechanisms that are important for CTGF mediated tumor growth do not depend on CTGF overexpression in tumor epithelial cells in MBC.

However, epithelial cell CTGF expression did correlate with a triple negative status of the tumor in FBC. All of the triple negative FBC also had stromal CTGF expression, most with high expression. This is an interesting finding and should be confirmed in a larger cohort. A compartment specific role for CTGF in tumor formation in FBC was proposed by Caparelli et al. as mentioned before. In their study using a triple negative breast cancer cell line, overexpression of CTGF by tumor epithelial cells lead to tumor cell digestion and inhibition of tumor growth, and overexpression of CTGF by fibroblast supports tumor growth. In the light of our results it would be interesting to consider and analyze the therapeutic potential of anti-CTGF therapy in triple negative FBC. In MBC epithelial cell CTGF expression did not correlate with a triple negative status, which could be due to the low number of triple negative MBC (n = 5).

It is a matter of debate what the best method is to analyze CTGF expression. IHC may have may its limitations, but it does allow selective analysis of expression in the stromal and epithelial compartments. In the dataset GSE31259 [22], there is no correlation between CTGF gene expression and grade, possibly related to the fact that CTGF expression in both stroma and epithelium is measured.

CTGF expression in MBC was not associated with survival in univariate survival analysis, despite the correlation between high stromal expression and high grade and high proliferation. This is in line with the lack of prognostic of CTGF in FBC, although also in FBC some correlations with established prognostic variables have been described [5].

In conclusion, stromal CTGF expression was seen in a high percentage of MBC and was correlated with high grade and high proliferation index. Expression in tumor epithelial cells was seen in a much lower percentage of cases. In view of the important role of the microenvironment in cancer progression, this makes stromal CTGF an interesting target for novel therapies and molecular imaging, but as a prognosticator. The role of CTGF as a therapeutic target for triple negative FBC deserves to be further studied.

## References

[1] Bork P. The modular architecture of a new family of growth regulators related to connective tissue growth factor. FEBS Lett. 1993 7 26;327(2):125–30. 7687569 10.1016/0014-5793(93)80155-n

[2] Chien W, O'Kelly J, Lu D, Leiter A, Sohn J, Yin D, et al Expression of connective tissue growth factor (CTGF/CCN2) in breast cancer cells is associated with increased migration and angiogenesis. Int J Oncol. 2011 6; 38(6):1741–7. 10.3892/ijo.2011.985 21455569 PMC3711677

[3] Perbal B. CCN proteins: A centralized communication network. J Cell Commun Signal. 2013 8; 7(3):169–77. 10.1007/s12079-013-0193-7 23420091 PMC3709049

[4] Yeger H, Perbal B. The CCN family of genes: a perspective on CCN biology and therapeutic potential. J Cell Commun Signal. 2007 12; 1(3–4):159–64. 10.1007/s12079-007-0005-z 18568428 PMC2443235

[5] Xie D, Nakachi K, Wang H, Elashoff R, Koeffler HP. Elevated levels of connective tissue growth factor, WISP-1, and CYR61 in primary breast cancers associated with more advanced features. Cancer Res. 2001 12 15; 61(24):8917–23. 11751417

[6] Shaaban AM, Ball GR, Brannan RA, Cserni G, Di Benedetto A, Dent J, et al A comparative biomarker study of 514 matched cases of male and female breast cancer reveals gender-specific biological differences. Breast Cancer Res Treat. 2012 6; 133(3):949–58. 10.1007/s10549-011-1856-9 22094935

[7] Kornegoor R, Verschuur-Maes AH, Buerger H, Hogenes MC, de Bruin PC, Oudejans JJ, et al Molecular subtyping of male breast cancer by immunohistochemistry. Mod Pathol. 2012 3; 25(3):398–404. 10.1038/modpathol.2011.174 22056953

[8] Kornegoor R, Moelans CB, Verschuur-Maes AH, Hogenes MC, de Bruin PC, Oudejans JJ, et al Promoter hypermethylation in male breast cancer: analysis by multiplex ligation-dependent probe amplification. Breast Cancer Res. 2012 7 5; 14(4):R101 10.1186/bcr3220 22765268 PMC3680933

[9] Kornegoor R, Verschuur-Maes AH, Buerger H, Hogenes MC, de Bruin PC, Oudejans JJ, et al Fibrotic focus and hypoxia in male breast cancer. Mod Pathol. 2012 6 8. 10.1038/modpathol.2012.101 22684218

[10] Kornegoor R, Moelans CB, Verschuur-Maes AH, Hogenes MC, de Bruin PC, Oudejans JJ, et al Oncogene amplification in male breast cancer: analysis by multiplex ligation-dependent probe amplification. Breast Cancer Res Treat. 2012 8; 135(1):49–58. 10.1007/s10549-012-2051-3 22527098 PMC3413821

[11] Weber-Chappuis K, Bieri-Burger S, Hurlimann J. Comparison of prognostic markers detected by immunohistochemistry in male and female breast carcinomas. Eur J Cancer. 1996 9; 32A(10):1686–92. 8983275 10.1016/0959-8049(96)00154-2

[12] Lacle MM, Kornegoor R, Moelans CB, Maes-Verschuur AH, van der Pol C, Witkamp AJ, et al Analysis of copy number changes on chromosome 16q in male breast cancer by multiplex ligation-dependent probe amplification. Mod Pathol. 2013 11; 26(11):1461–7. 10.1038/modpathol.2013.94 23743929

[13] Bocker W. WHO classification of breast tumors and tumors of the female genital organs: pathology and genetics. Verh Dtsch Ges Pathol. 2002; 86:116–9. 12647359

[14] Central Committee on Research involving Human Subjects.

[15] van Diest PJ. No consent should be needed for using leftover body material for scientific purposes. For. BMJ. 2002 9 21; 325(7365):648–51. 12242180

[16] Lacle MM, van der Pol C, Witkamp A, van der Wall E, van Diest PJ. Prognostic value of mitotic index and Bcl2 expression in male breast cancer. PLOS One.8(4):e60138 10.1371/journal.pone.0060138 23573235 PMC3613416

[17] Boersema M, Katta K, Rienstra H, Molema G, Nguyen TQ, Goldschmeding R, et al Local medial microenvironment directs phenotypic modulation of smooth muscle cells after experimental renal transplantation. Am J Transplant. 2012 6; 12(6):1429–40. 10.1111/j.1600-6143.2012.04001.x 22420764

[18] Frazier KS, Grotendorst GR. Expression of connective tissue growth factor mRNA in the fibrous stroma of mammary tumors. Int J Biochem Cell Biol. 1997 1; 29(1):153–61. 9076950 10.1016/s1357-2725(96)00127-6

[19] Capparelli C, Whitaker-Menezes D, Guido C, Balliet R, Pestell TG, Howell A, et al CTGF drives autophagy, glycolysis and senescence in cancer-associated fibroblasts via HIF1 activation, metabolically promoting tumor growth. Cell Cycle. Jun 15;11(12):2272–84. 10.4161/cc.20717 22684333 PMC3383589

[20] Xiu M, Liu YH, Brigstock DR, He FH, Zhang RJ, Gao RP. Connective tissue growth factor is overexpressed in human hepatocellular carcinoma and promotes cell invasion and growth. World J Gastroenterol. 2012 12 21; 18(47):7070–8. 10.3748/wjg.v18.i47.7070 23323010 PMC3531696

[21] Jia XQ, Cheng HQ, Li H, Zhu Y, Li YH, Feng ZQ, et al Inhibition of connective tissue growth factor overexpression decreases growth of hepatocellular carcinoma cells in vitro and in vivo. Chin Med J (Engl). 2011 11; 124(22):3794–9. 22340243

[22] Johansson I, Nilsson C, Berglund P, Lauss M, Ringner M, Olsson H, et al Gene expression profiling of primary male breast cancers reveals two unique subgroups and identifies N-acetyltransferase-1 (NAT1) as a novel prognostic biomarker. Breast Cancer Res.14(1):R31 22333393 10.1186/bcr3116 PMC3496149

